# Diagnostic accuracy of dual-energy CT in distinguishing intracerebral hemorrhage from contrast staining: a systematic review and meta-analysis with radiation dose assessment

**DOI:** 10.1007/s11547-026-02180-6

**Published:** 2026-02-11

**Authors:** Luigi Asmundo, Moreno Zanardo, Domenico Albano, Mariachiara Basile, Massimo Cressoni, Attilio Prato, Francesco Sardanelli, Luca Maria Sconfienza, Paolo Vitali

**Affiliations:** 1 Department of Radiology, ASST Grande Ospedale Metropolitano Niguarda, Piazza Ospedale Maggiore 3, 20162 Milan, Italy; 2 Radiology Unit, IRCCS Policlinico San Donato, Via Morandi 30, San Donato Milanese, 20097 Milan, Italy; 3 Dipartimento di Scienze Biomediche, Chirurgiche ed Odontoiatriche, Università Degli Studi di Milano, Milan, Italy; 4 Radiology Unit, IRCCS Istituto Ortopedico Galeazzi, Milan, Italy; 5 Postgraduation School in Radiodiagnostics, Università Degli Studi di Milano, Via Festa del Perdono 7, 20122 Milan, Italy; 6 Lega Italiana per la lotta contro i tumori (LILT) Milano Monza Brianza, Milan, Italy; 7 Department of Biomedical Sciences for Health, Università Degli Studi di Milano, Via Mangiagalli 31, 20133 Milan, Italy

**Keywords:** Stroke, Intracerebral hemorrhage, Dual-energy, Contrast media, Diagnostic accuracy

## Abstract

**Purpose:**

This systematic review and meta-analysis aimed to investigate the diagnostic accuracy of dual-energy CT (DECT) in distinguishing intracerebral hemorrhage (ICH) from contrast staining after procedures and to assess the associated radiation dose in comparison with conventional CT.

**Methods:**

A systematic search was conducted up to December 2024. Eligible studies included those applying DECT for cerebrovascular conditions with retrievable technical and diagnostic performance data. Pooled estimates of sensitivity, specificity, and radiation dose parameters were calculated using a random-effects model.

**Results:**

A total of 68 studies, including 5530 patients, met the inclusion criteria. Among them, 34/68 (50%) focused on lesion detection, 18/68 (26%) on technical aspects, and 16/68 (24%) on prediction. A meta-analysis of 10 studies demonstrated a pooled sensitivity of 96.1% (95% CI 83.8%–99.1%) and specificity of 97.8% (95% CI 91.4%–99.5%) for differentiating ICH from contrast staining. Additionally, a radiation dose meta-analysis of 13 studies provided pooled estimates of computed tomography dose index volume (CTDIvol) at 28.83 mGy (95% CI 20.60–37.07 mGy) and dose-length product (DLP) at 517.66 mGy × cm (95% CI 400.19–635.13 mGy × cm), comparable to conventional single-energy CT.

**Conclusion:**

DECT demonstrates excellent diagnostic accuracy in differentiating ICH from contrast staining, with radiation exposure comparable to conventional CT. The large variability in voltage and doses among different protocols reflects the relative immaturity of DECT and the need for multicentric harmonization and standardization. Given its high diagnostic accuracy and comparable radiation exposure to single-energy CT, where technically available, DECT should always be considered in the specific scenario of differentiating ICH from contrast staining.

**Clinical relevance statement:**

DECT provides high diagnostic accuracy without increasing radiation exposure, enabling confident post-treatment differentiation between hemorrhage and contrast staining to guide timely therapeutic decisions.

**Supplementary Information:**

The online version contains supplementary material available at 10.1007/s11547-026-02180-6.

## Introduction

Computed tomography (CT) is crucial for diagnosing acute cerebrovascular disease, particularly strokes, which remain a leading cause of mortality and morbidity worldwide [1]. Stroke, either ischemic or hemorrhagic, leads to irreversible brain damage.

Acute stroke CT protocols typically include non-contrast CT [2], CT angiography, and perfusion imaging [3–5], which can be, respectively, used to differentiate between ischemic and hemorrhagic stroke, in ischemic stroke to identify large vessel occlusion and to assess eligibility for mechanical or pharmacological thrombolysis [2]. Non-contrast CT is the first-line imaging modality for suspected strokes due to its speed, availability, and ability to exclude hemorrhage while sometimes identifying early ischemic changes [6]. CT angiography and CT perfusion provide fundamental information about vascular status and salvageable brain tissue [5].

In recent years, dual-energy computed tomography (DECT) has gained attention for its potential applications in acute cerebrovascular disease. DECT has demonstrated utility in predicting outcomes such as acute cerebral infarction, intracerebral hemorrhage (ICH), and other complications in patients undergoing various stroke treatments [7, 8]. Beyond acute settings, DECT has also been applied to evaluate cerebral arteries in patients with transient ischemic attacks (TIA) and chronic cerebrovascular conditions.

**Fig. 1:**
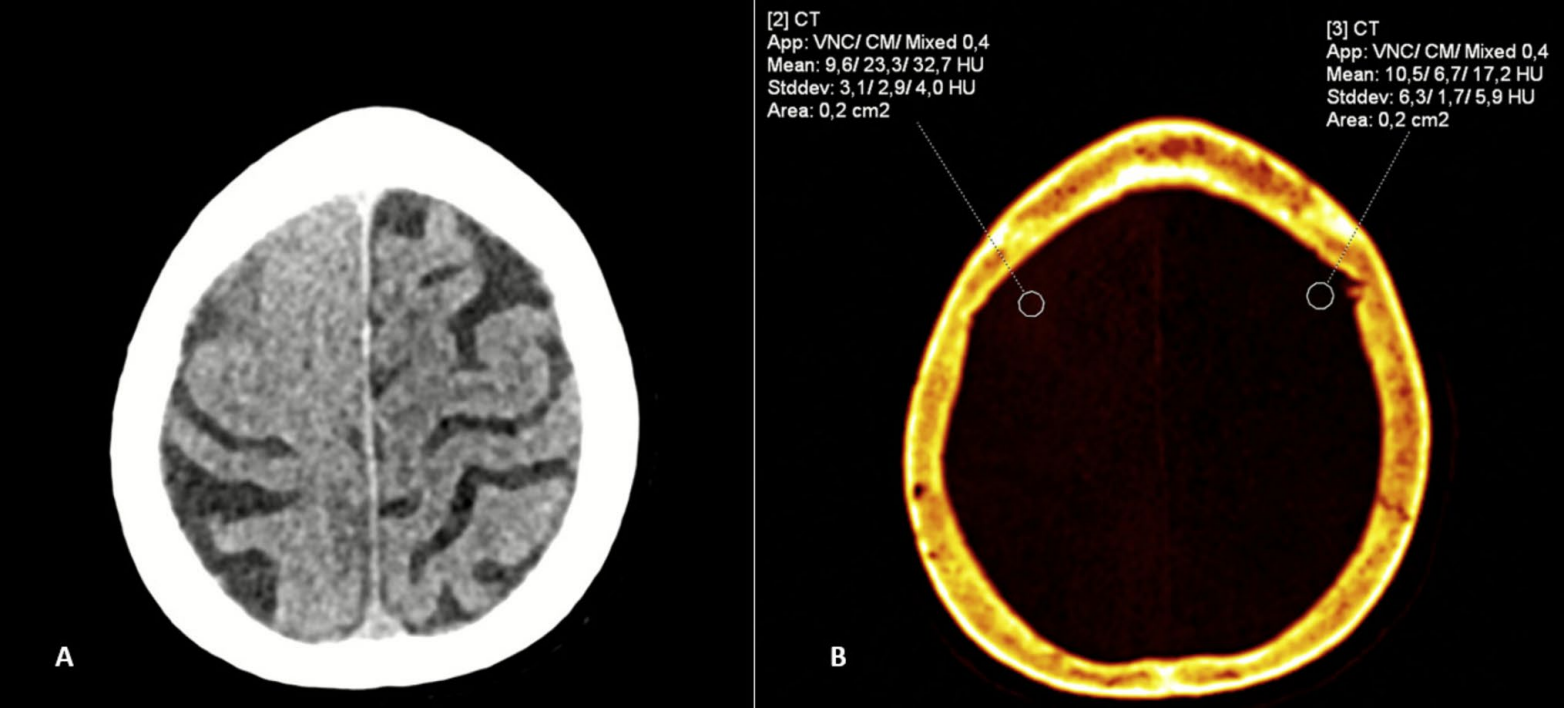
After a percutaneous coronary intervention, an 85 y-o woman presented mild left hemiparesis. Virtual MonoEnergetic (VME, 80 and 140 kV scans) image shows diffuse subarachnoid hyperdensity over the right hemisphere. Virtual non-contrast/contrast media (VNC/CM) image at the same level shows that hyperdensity (32.7 HU) is due to contrast extravasation (CM: 23.3 HU) and not to subarachnoid hemorrhage (VNC: 9.6 HU). Imaging by IRCCS Policlinico San Donato, Milan, Italy, by Siemens Somatom Definition Flash 256 slices scanner and Syngo CT dual-energy tool

A notable advantage of DECT is its ability to distinguish between hemorrhagic transformation and contrast staining after procedures. By generating iodine maps (IMs) and virtual non-contrast (VNC) images, DECT can identify regions of hyper-attenuation due to contrast rather than blood, providing critical diagnostic clarity in the post-treatment setting. In fact, iodine overlay maps reveal the presence of contrast, while VNC images confirm the absence of hyper-attenuation when it is attributable to contrast, thereby ruling out hemorrhage (Fig. 1). Thus, DECT offers promising benefits in the diagnostic evaluation of cerebrovascular diseases. Although previous systematic reviews and meta-analyses have investigated the role of DECT in acute cerebrovascular disease, these studies were either limited in scope, focused primarily on small subsets of patients or provided aggregate findings without separately quantifying diagnostic performance for the critical differentiation between ICH and contrast staining. Moreover, many of these earlier reviews did not address radiation dose implications or technical acquisition parameters in a systematic way. In recent years, several additional studies have been published, reflecting technological advances in DECT scanners and post-processing algorithms. An updated and comprehensive synthesis is therefore needed. The present systematic review and meta-analysis aims to fill this gap by (i) providing updated pooled estimates of DECT diagnostic accuracy specifically for distinguishing ICH from contrast staining; (ii) analyzing radiation dose parameters in comparison with conventional CT; and (iii) summarizing technical protocols and post-processing approaches across studies. This broader scope allows for a more complete appraisal of DECT’s current clinical utility and future potential in cerebrovascular imaging.

## Materials and methods

No ethics committee approval was needed to perform this systematic review. We registered our systematic review and meta-analysis on Prospero (CRD42021238825, https://www.crd.york.ac.uk/prospero/display_record.php?RecordID=238825), and it was reported according to the Preferred Reporting Items for Systematic Reviews and Meta-Analyses (PRISMA) [9] and PRISMA for Diagnostic Test Accuracy [10].

### Study design

In December 2024, a systematic search was conducted on MEDLINE (PubMed) and EMBASE (Elsevier), for studies reporting the use of DECT for clinical applications of the brain. The search was limited to original studies written in English, published either on paper or online on peer-reviewed journals, with an available abstract. We used extensive search terms to include all possible applicable studies. In addition, the included articles’ references were manually searched for possibly relevant research. Full search strategies are reported in Supplementary Material.

Three researchers (L.A., M.Z., and A.P., with, respectively, 4, 3, and 3 years of experience in neuroimaging) performed in consensus an initial screening of the retrieved articles, excluding reviews, case reports, and studies that only comprised automatic computer analyses. A consensus-based approach was adopted to ensure methodological consistency and minimize individual bias through direct discussion and agreement at each step*.* Full text was downloaded for all studies included at this first selection, and a second screening was performed. Finally, references of included articles and reviews were manually searched to check for further eligible studies.

We included a study if: 1) DECT was used on humans; 2) DECT was used for cerebrovascular-related pathologies; 3) DECT technical data were retrievable, or if these data were derivable from the presented tables or supplementary materials. Exclusion criteria were: 1) studies with patients not referred to a DECT or results not provided and 2) studies in vitro or on animal models.

### Data extraction

For all articles included in the final selection, data extraction was performed by the same researchers who conducted the selection process. In cases of disagreement among the reviewers, arbitration was resolved through consensus. For each study, the extracted data included (when available) the year of publication, continent and country of publication, study design (prospective or retrospective), number of patients, number and percentage of male patients, patients’ mean age, DECT system, detector rows, DECT method, low and high kVp, high and low mAs, contrast agent type, iodine concentration, dose and flow rate, radiation dose, and post-processing methods used for DECT. Additionally, information on the main study endpoint, the use of perfusion techniques, and relevant technical details were extracted. Each included study was further divided into subgroups based on the number of different post-processing methods employed for DECT. Finally, if available, key outcome measures such as true positives, false positives, true negatives, and false negatives were retrieved. These data were used to calculate sensitivity, specificity, and diagnostic accuracy, facilitating the performance of a diagnostic test accuracy meta-analysis.

### Quality assessment

Another researcher (M.B.) with 4 years’ experience in neuroimaging reviewed the quality of the included articles, using the QualSyst tool [11], using the checklist for qualitative studies, and QUADAS 2 tool [12] for only the diagnostic test accuracy (DTA) articles included.

### Diagnostic performance measures and statistical analysis

For the meta-analysis, only studies reporting complete diagnostic contingency data (true positives, false positives, true negatives, and false negatives) or sufficient information to derive these parameters were included. Studies reporting only qualitative or descriptive results aggregate performance metrics without raw data, or primarily technical evaluations without diagnostic accuracy endpoints were excluded from the quantitative synthesis and summarized narratively. Pooled sensitivity and specificity were computed using data on the numbers of true-positive, false-negative, true-negative, and false-positive findings. The sensitivity and specificity of DECT in distinguishing ICH from contrast staining were calculated for each study included in the DTA analysis using a bivariate random-effects model to estimate pooled data. The summary receiver operating characteristic (sROC) curve was used to calculate the area under the curve (AUC), providing a comprehensive measure of diagnostic performance. Sensitivity and specificity are presented along with their corresponding 95% confidence intervals (CIs) in a forest plot, generated using Meta-DiSc 2.0 [13–18]. For studies reporting 100% sensitivity or specificity (zero-cell studies), a continuity correction of 0.5 was applied to all cells of the 2 × 2 table before pooling, in line with recommendations for bivariate random-effects DTA meta-analyses.

The meta-analysis to obtain estimated pooled computed tomography dose index volume (CTDI*vol)* and dose-length product (DLP) was conducted according to the random-effects model, using the DerSimonian–Laird method [19]. Heterogeneity was assessed using Cochran’s *Q* and the *I*^2^ statistic, which quantifies the proportion of total variation due to heterogeneity rather than chance. An *I*^2^ value between 50 and 75% indicates substantial heterogeneity, while values above 75% suggest considerable heterogeneity [20, 21]. Because the included studies encompassed diverse DECT protocols (non-contrast, angiographic, and perfusion imaging), pooled CTDIvol and DLP estimates were calculated inclusively to reflect overall radiation exposure in cerebrovascular DECT applications. This approach prioritized comprehensive representation of current clinical practice over protocol-specific stratification, which would have limited comparability across studies.

## Results

### Search outcomes

**Fig. 2:**
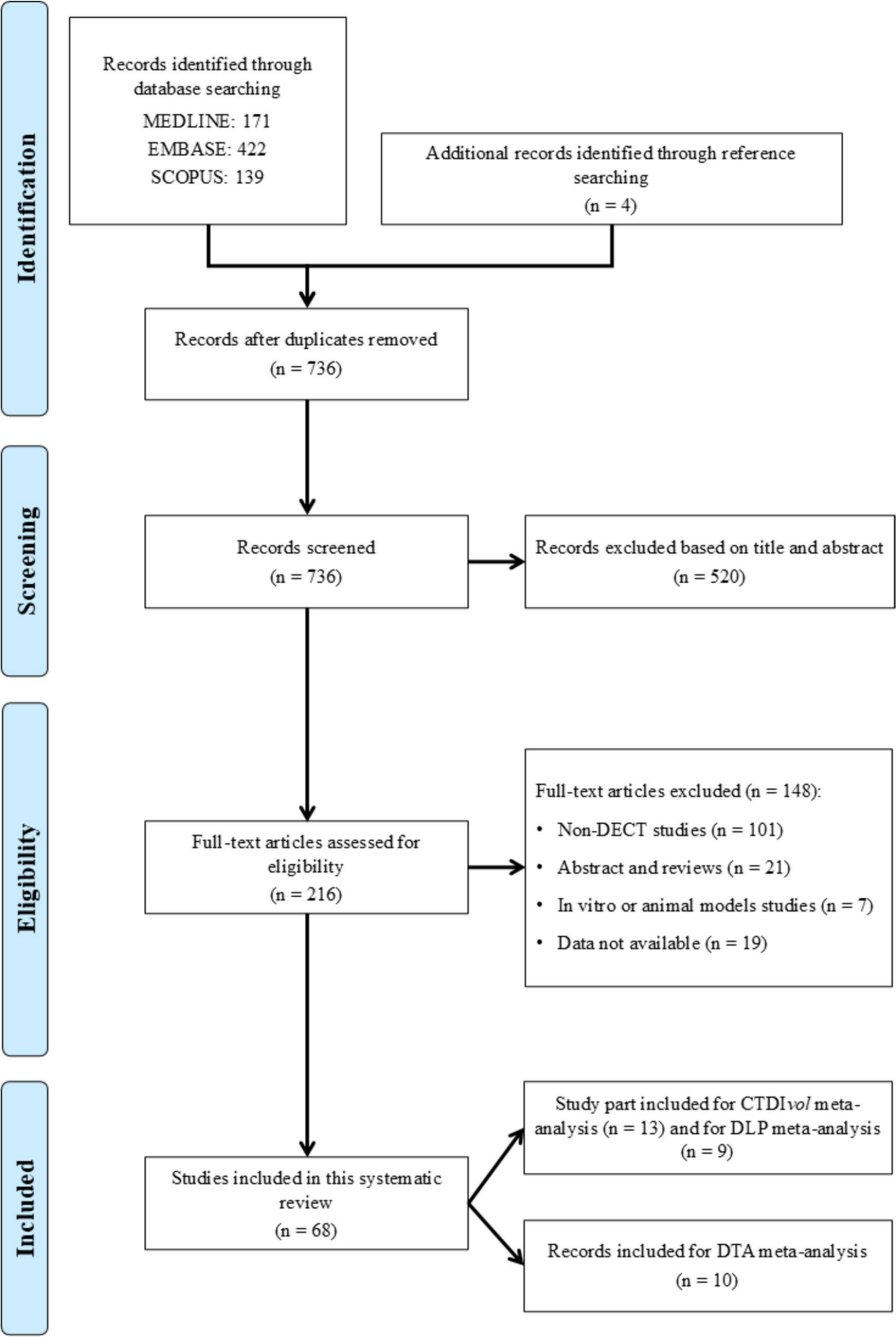
Flowchart depicting the study selection process, according to the Preferred Reporting Item for Systematic Reviews and Meta-Analyses (PRISMA) [9]

Starting from 732 records identified through search query and four identified through other sources such as references from included works, 520 were excluded from title and abstract, while the remaining 216 were downloaded for individual assessment. Finally, a total of 68 articles matched the inclusion criteria [8, 22–88]. A flowchart of study selection is shown in Fig. 2.

Included articles were published between 2008 and 2024. Study design was retrospective in 55/68 (81%) papers and prospective in 13/68 papers (19%). The most represented countries in the included studies are from China (15/68, 22%), USA (14/68, 21%), and the Netherlands (5/68, 7%). Study population ranged from 6 to 424 patients, for a total of 5530 enrolled patients. Patients’ ages ranged from 38 to 76 years.

Studies included in the analysis reported a range of endpoints related to the clinical applications of DECT, including diagnostic accuracy for acute ischemic stroke, prognostic markers such as iodine concentration as an indicator of blood–brain barrier disruption, and evaluation of post-treatment complications following endovascular therapy or intravenous thrombolysis, including cerebral arterial air emboli, hemorrhagic transformation, and contrast extravasation. Additional endpoints focused on image quality and comparisons of DECT reconstructions, such as VNC images, IMs, and virtual monoenergetic (VM) imaging, with standard imaging techniques, assessing quality, artifact reduction, clinical applicability, and technical feasibility.

Half of the studies (34/68, 50%) focused on the role of DECT in lesion detection, followed by studies on technical aspects (18/68, 26%) and on prediction of hemorrhagic transformation in ischemic stroke (16/68, 24%). Among analyzed studies, 23/68 (34%) specifically addressed the differentiation between contrast staining and ICH.

### DECT technology and acquisition parameters

DECT technology was dual-source in 58/68 (85%) articles, fast kVp-switching in 6/68 (9%) articles, spectral detector in 2/68 (3%) articles, and single-source in 2/68 (3%). The most used DECT systems included scanners from Siemens in 58/68 (85%) articles, while General Electric in 6/68 (9%) articles and Philips in 4/68 (6%) articles.

Acquisition protocols varied among studies, incorporating combinations of low-energy and high-energy tube voltage (kVp), with the most frequent combination of 80 low-kVp and 140 high-kVp used in 34/68 (50%) articles, with a tube current extremely variable (minimum 35–maximum 800 mAs) tailored to the clinical application and study context.

Information on iodinated contrast agents was reported in 22/68 (32%) of studies, with details on concentration (300 mgI/mL adopted in 7/22 articles) and dosage (mainly fixed amount ranging from 40 to 100 mL).

Post-processing techniques included VNC images reported in 37/68 (54%) articles, IMs in 28/68 (41%), and virtual monochromatic or monoenergetic images in 16/68 (24%), while other advanced methods were less frequently reported (18/68, 26%, mainly bone removal application or X-maps).

Technical data from the included studies are reported in Tables 1, 2 and 3.

**Table 1:** Extracted data from the studies and study parts included in the review including study design, patient demographics, CT scanner models, dual-energy (DE) CT methods, imaging parameters (kVp mAs), and contrast agent details (dose and iodine concentration)

Study ID	Country	Study design	Patients	Age (years)	Age SD or range (years)	CT scanner	CT rows	DE method
Cui 2024	China	Retrospective	53	67	32–84	Somatom Definition Force (Siemens Healthcare)	128	Dual-source
Robbe 2024	Netherlands	Retrospective	194	75	64–83	Somatom Definition Flash (Siemens Healthcare)	128	Dual-source
Coorens 2023	Usa	Retrospective	107	66	17	Somatom Definition Force (Siemens Healthcare)	192	Dual-source
Dai 2023	China	Retrospective	86	71	60–77	GE Revolution CT (GE Healthcare)	256	Fast kVp-switching
Grkovski 2023	Switzerland	Retrospective	41	73	11.2	Somatom X.cite (Siemens Healthcare)	128	Single-source
Huijberts 2023	Netherlands	Retrospective	402	73	63–82	Somatom Definition Flash/Force, Siemens Healthcare, Forchheim Germany	192	Dual-source
Jiang 2023	China	Retrospective	88	66	12.46	Somatom Drive (Siemens Healthcare)	192	Dual-source
Pinckaers 2023	Netherlands	Prospective	214	71	63–81	Somatom Definition Flash/Force, Siemens Healthcare, Forchheim Germany	256	Dual-source
Chrzan 2022	Poland	Retrospective	64	69	28–90	GE Revolution CT (GE Healthcare)	256	Fast kVp-switching
Dodig 2022	Croatia	Prospective	184	71	62–80	Somatom Definition Flash (Siemens Healthcare)	256	Dual-source
Grkovski 2022	Switzerland	Retrospective	39	69	11	Somatom X.cite (Siemens Healthcare)	128	Single-source
Kauw 2022	Usa	Retrospective	193	67	16	Somatom Definition Force (Siemens Healthcare)	192	Dual-source
Ma 2022	China	Retrospective	138	69	62–77	Somatom Definition Force (Siemens Healthcare)	192	Dual-source
Renu 2022	Spain	Retrospective	424	73	63–81	Somatom Definition Flash (Siemens Healthcare)	64	Dual-source
Chen 2021	China	Retrospective	46	67	7.8	Somatom Definition Force (Siemens Healthcare)	192	Dual-source
Fransson 2021	Sweden	Retrospective	10		61–97	IQon Spectral CT (Philips Healthcare)	64	Dual-layer
Riederer 2021	Germany	Retrospective	47	72	15	IQon Spectral CT (Philips Healthcare)	64	Dual-layer
Liu 2021	China	Retrospective	106	72	10	Siemens Healthcare (unspecified)	128	Dual-source
van den Broek 2021	Canada	Retrospective	51	76	67–82	Somatom Definition Flash (Siemens Healthcare)	128	Dual-source
van Ommen 2021	Usa	Retrospective	25	70	21–87	Philips Healthcare (unspecified)		Dual-layer
Wang 2021	China	Prospective	44	66	9.6	Somatom Definition Flash (Siemens Healthcare)	256	Dual-source
Wolman 2021	Usa	Retrospective	24	64	15	Somatom Definition Flash (Siemens Healthcare)	256	Dual-source
Almqvist 2020	Sweden	Retrospective	168	71	59–80	Discovery HD 750 (GE Healthcare)	64	Fast kVp-switching
Byrne 2020	Usa	Retrospective	71	70	18–97	Somatom Definition Flash/Force (Siemens Healthcare)		Dual-source
Cai 2020	China	Retrospective	147	71	11	Somatom Definition Flash (Siemens Healthcare)	128	Dual-source
Liu 2020	China	Retrospective	106	72	10	Somatom Definition Force (Siemens Healthcare)	192	Dual-source
Ma 2020	China	Retrospective	102	70	13	Somatom Definition Force (Siemens Healthcare)	192	Dual-source
Murias 2020	Spain	Retrospective	50	68	19	Somatom Definition Flash (Siemens Healthcare)	128	Dual-source
Ståhl 2020	Sweden	Retrospective	24	75	65–82	IQon Spectral CT (Philips Healthcare)	64	Dual-layer
van Ommen 2020	Netherlands	Retrospective	125	65	52–80	Somatom Definition Flash (Siemens Healthcare)	128	Dual-source
Wiggins 2020	Usa	Retrospective	137	66	54–78	Somatom Definition Flash (Siemens Healthcare)		Dual-source
Zaouak 2020	Belgium	Prospective	35	55	44–66	Somatom Definition Force (Siemens Healthcare)	192	Dual-source
Almqvist 2019	Sweden	Retrospective	372	69	60–77	Discovery HD 750 (GE Healthcare)		Fast kVp-switching
An 2019	China	Prospective	180	61	13	Somatom Definition Flash (Siemens Healthcare)		Dual-source
Bodanapally 2019	Usa	Retrospective	65	48	25–66	Somatom Definition Force (Siemens Healthcare)	64	Dual-source
Ebashi 2019	Japan	Retrospective	52	75	13	Somatom Definition Force (Siemens Healthcare)	256	Dual-source
Tan 2019	Usa	Retrospective	42	66	15	Somatom Definition Force (Siemens Healthcare)	64	Dual-source
Yun 2019	South Korea	Retrospective	76	61	9	Somatom Drive (Siemens Healthcare)	192	Dual-source
Zaouak 2019	Belgium	Prospective	35	55	44–66	Somatom Definition Force (Siemens Healthcare)	192	Dual-source
Bodanapally 2018	Usa	Retrospective	40	38	18–73	Somatom Definition Force (Siemens Healthcare)		Dual-source
Bonatti 2018	Italy	Retrospective	85	70	31–87	Somatom Definition Flash (Siemens Healthcare)	128	Dual-source
Grams 2018	Austria	Retrospective	46	63	24–89	Somatom Definition Flash (Siemens Healthcare)		Dual-source
Mocanu 2018	Belgium	Prospective	35	58	35–78	Somatom Definition Force (Siemens Healthcare)	192	Dual-source
Taguchi 2018	Usa	Prospective	11	76	68–90	Somatom Definition Force (Siemens Healthcare)	192	Dual-source
Bodanapally 2017	Usa	Retrospective	24	69	60–78	Somatom Definition Force (Siemens Healthcare)	192	Dual-source
Leithner 2017	Germany	Retrospective	40	62	17.0	Somatom Definition Force (Siemens Healthcare)	192	Dual-source
Naruto 2018	Japan	Retrospective	16	64	18–90	Somatom Definition Force (Siemens Healthcare)	192	Dual-source
Scholtz 2017	Germany	Retrospective	55	68	19.9	Somatom Definition Force (Siemens Healthcare)	192	Dual-source
Winklhofer 2017	Switzerland	Retrospective	30	68	36–90	Somatom Definition Flash (Siemens Healthcare)	32	Dual-source
Bonatti 2016	Italy	Retrospective	134	64	18–93	Somatom Definition Flash (Siemens Healthcare)	128	Dual-source
Djurdjevic 2016	Austria	Retrospective	20	64	14.99	Somatom Definition Flash (Siemens Healthcare)	128	Dual-source
Hixson 2016	Usa	Retrospective	30	64	22–93	Discovery HD 750 (GE Healthcare)	32	Fast kVp-switching
Noguchi 2016	Japan	Retrospective	6	70	58–76	Somatom Definition Force (Siemens Healthcare)	192	Dual-source
Wang 2016	China	Retrospective	30	62	28–80	Somatom Definition Flash (Siemens Healthcare)	128	Dual-source
Yang 2016	China	Retrospective	40	56	12.40	Somatom Definition Flash (Siemens Healthcare)	128	Dual-source
Gariani 2015	Switzerland	Retrospective	58	70	33–91	Somatom Definition Flash (Siemens Healthcare)	128	Dual-source
Grams 2015	Austria	Prospective	16	64	13.09	Somatom Definition Flash (Siemens Healthcare)	128	Dual-source
Guo 2015	China	Retrospective	9	42	28–64	Siemens Healthcare (unspecified)		Dual-source
Renú 2015	Spain	Prospective	132	68	14	Somatom Definition Flash (Siemens Healthcare)	64	Dual-source
Watanabe 2014	Japan	Retrospective	36	60	16–84	Somatom Definition Flash (Siemens Healthcare)	128	Dual-source
Morhard 2013	Germany	Retrospective	60	73	12.3	Somatom Definition Flash (Siemens Healthcare)	256	Dual-source
Shinohara 2013	Japan	Retrospective	13	63	37–80	Discovery HD 750 (GE Healthcare)	64	Fast kVp-switching
Tijssen 2013	Netherlands	Retrospective	22	56	16.4	Somatom Definition Flash (Siemens Healthcare)	128	Dual-source
Phan 2012	Usa	Prospective	40	65	28–94	Somatom Definition (Siemens Healthcare)	64	Dual-source
Gupta 2010	Usa	Retrospective	18	64	36–93	Somatom Definition Flash (Siemens Healthcare)	64	Dual-source
Zhang 2010	China	Prospective	80	52	9	Somatom Definition Flash (Siemens Healthcare)	64	Dual-source
Ferda 2009	Czech Republic	Retrospective	25	53	25–75	Somatom Definition Flash (Siemens Healthcare)	64	Dual-source
Watanabe 2008	Japan	Prospective	12	64	36–78	Somatom Definition Flash (Siemens Healthcare)	64	Dual-source

Study ID	Low/High-energy kVp	Low/High-energy mAs	Contrast agent	Iodine concentration (mgI/mL)	Total dose (mL)	Dose perfusion (mL)	Dose (mL/kg)
Cui 2024	80/150	800/533					
Robbe 2024	80/140	500/250	Iopromide	300	50		
Coorens 2023	80/140						
Dai 2023	80/140		Iopamidol	370			1
Grkovski 2023	80/150	318/379					
Huijberts 2023	80/140	392/196					
Jiang 2023	90/150	69/90	Iopromide	370			1.5
Pinckaers 2023	80/140	196/392					
Chrzan 2022	80/140	315/375					
Dodig 2022	80/140	270/138					
Grkovski 2022	80/150	179/220					
Kauw 2022	80/140	640/320					
Ma 2022	80/150	310/207					
Renu 2022	100/140	250/250					
Chen 2021	80/150	122/68		320	6		
Fransson 2021	120/140						
Riederer 2021	120	261	Iomeprol				
Liu 2021	80/140	392/196					
van den Broek 2021	90/150		Iohexol	350	70		
van Ommen 2021	120	35	Iopromide	300	50		
Wang 2021	80/150						
Wolman 2021	80/140	640/320					
Almqvist 2020	80/140						
Byrne 2020	80/140						
Cai 2020	80/140						
Liu 2020	80/140	392/196					
Ma 2020	80/150	310/207					
Murias 2020							
Ståhl 2020	120/120	254/254					
van Ommen 2020	80/140	640/320					
Wiggins 2020	100/140	300/300					
Zaouak 2020	80/150	310/207					
Almqvist 2019	80/140						
An 2019	90/150						
Bodanapally 2019	80/150	273/410	Iohexol	350	100		
Ebashi 2019	80/150						
Tan 2019	80/140	499/118					
Yun 2019	80/140	300/150	Iohexol	350	50		
Zaouak 2019	80/150	310/207					
Bodanapally 2018	80/150	410/273	Iohexol	300	100		
Bonatti 2018	80/140	310/155					
Grams 2018	100/140	360/360					
Mocanu 2018	80/150	100/67	Iomeron	400	60		
Taguchi 2018	80/150	800/533					
Bodanapally 2017	80/150	410/273	Iohexol	350	100		
Leithner 2017	90/150	95/59	Iopromide	300	90		0.9
Naruto 2018	80/150	800/533					
Scholtz 2017	80/150	410/273					
Winklhofer 2017	80/140	222/111	Iobitridol	350	80		
Bonatti 2016	80/140	222/111	Iobitridol	350	75		
Djurdjevic 2016	100/140	324/324					
Hixson 2016	80/140						
Noguchi 2016	80/150	800/533					
Wang 2016	80/140	177/89	Iohexol	350	40		
Yang 2016	80/140	270/135	Iopromide	370	60		
Gariani 2015	80/140						
Grams 2015	100/140	360/360					
Guo 2015	80/140	230/50	Iopamidol	370	60–75		
Renú 2015	100/140	250/250	Iopromide	300			
Watanabe 2014	100/140	360/80	Unspecified	300	100		
Morhard 2013	80/140	310/155	Unspecified				
Shinohara 2013	80/140	375/375	Iopamidol	370	50		
Tijssen 2013	80/140	392/196					
Phan 2012	80/140	499/118					
Gupta 2010	80/140	499/118					
Zhang 2010	80/140	360/51	Iopromide	300	80		
Ferda 2009	80/140	250/56		400	60		
Watanabe 2008	80/140	360/80		350	60–70-80

**Table 2:** Radiation dose metrics extracted from the studies, including computed tomography dose index volume (CTDIvol) and dose-length product (DLP), along with their respective standard deviations (SD) or ranges, when available

Study ID	CTDIvol (mGy)	SD or range CTDIvol (mGy)	DLP (mGy*cm)	SD or range DLP (mGy*cm)
Cui 2024				
Robbe 2024				
Coorens 2023				
Dai 2023				
Grkovski 2023	43.6	3.7		
Huijberts 2023	37			
Jiang 2023				
Pinckaers 2023	37			
Chrzan 2022	60			
Dodig 2022	25		418.5	
Grkovski 2022	43.7	3.4		
Kauw 2022				
Ma 2022				
Renu 2022				
Chen 2021				
Fransson 2021				
Riederer 2021	44.7			
Liu 2021				
van den Broek 2021	63.7	60.7–67.2	1060	981.0–1151.5
van Ommen 2021	6			
Wang 2021				
Wolman 2021				
Almqvist 2020	57			
Byrne 2020				
Cai 2020				
Liu 2020				
Ma 2020				
Murias 2020				
Ståhl 2020	48.2			
van Ommen 2020	59.8			
Wiggins 2020				
Zaouak 2020				
Almqvist 2019	57			
An 2019				
Bodanapally 2019				
Ebashi 2019				
Tan 2019	28.2	8.7		
Yun 2019	15.4	1.1	280.2	27.08
Zaouak 2019	21.72		400	
Bodanapally 2018				
Bonatti 2018				
Grams 2018				
Mocanu 2018	13	4–24	459	127–959
Taguchi 2018	80.08			
Bodanapally 2017	31.45	2.95	609.62	76.79
Leithner 2017				
Naruto 2018				
Scholtz 2017	41	0	770.6	90.2
Winklhofer 2017	18.3	2.96		
Bonatti 2016	19.3	0.02	372	37
Djurdjevic 2016				
Hixson 2016				
Noguchi 2016	80.08			
Wang 2016	6.53	0.23	120	11.67
Yang 2016	20			
Gariani 2015	61.84			
Grams 2015				
Guo 2015				
Renú 2015				
Watanabe 2014				
Morhard 2013	24.3			
Shinohara 2013				
Tijssen 2013	37		568	
Phan 2012	66			
Gupta 2010				
Zhang 2010	20.6	0.1	398.6	19
Ferda 2009	11.1			
Watanabe 2008

**Table 3:** Post-processing techniques used in the included studies, categorized by topic (Detection, Technical, Prediction)

Study ID	Topic	Virtual non-contrast (VNC)	Iodine maps (IM)	Virtual monochromatic images or virtual monoenergetic images (VMIs)	Others
Cui 2024	Detection				X
Robbe 2024	Technical				
Coorens 2023	Technical	X			
Dai 2023	Prediction		X	X	
Grkovski 2023	Technical	X			
Huijberts 2023	Prediction				X
Jiang 2023	Technical				
Pinckaers 2023	Prediction	X	X		
Chrzan 2022	Technical		X		
Dodig 2022	Detection				X
Grkovski 2022	Technical	X			
Kauw 2022	Detection	X			
Ma 2022	Prediction	X			
Renu 2022	Prediction	X			
Chen 2021	Technical	X			
Fransson 2021	Detection		X		X
Riederer 2021	Detection	X	X		
Liu 2021	Prediction	X	X		
van den Broek 2021	Detection	X			
van Ommen 2021	Technical			X	
Wang 2021	Prediction	X	X		
Wolman 2021	Detection				X
Almqvist 2020	Detection				
Byrne 2020	Prediction	X	X		
Cai 2020	Prediction	X	X		
Liu 2020	Technical	X	X	X	
Ma 2020	Prediction	X	X		
Ståhl 2020	Detection			X	
van Ommen 2020	Detection			X	
Wiggins 2020	Detection				X
Zaouak 2020	Detection	X	X		
Almqvist 2019	Detection		X	X	X
An 2019	Prediction	X	X		
Bodanapally 2019	Prediction	X	X	X	
Ebashi 2019	Detection	X	X		
Murias 2020	Technical				
Tan 2019	Prediction				
Yun 2019	Technical	X			
Zaouak 2019	Detection	X	X		
Bodanapally 2018	Technical				
Bonatti 2018	Prediction	X	X	X	
Grams 2018	Detection	X			X
Mocanu 2018	Technical			X	
Taguchi 2018	Technical				X
Bodanapally 2017	Detection	X	X	X	
Leithner 2017	Technical			X	
Naruto 2018	Detection			X	X
Scholtz 2017	Technical				X
Winklhofer 2017	Detection	X			
Bonatti 2016	Detection	X			
Djurdjevic 2016	Prediction	X	X		X
Hixson 2016	Detection			X	
Noguchi 2016	Detection				X
Wang 2016	Technical				X
Yang 2016	Detection				X
Gariani 2015	Detection	X			
Grams 2015	Detection	X	X		
Guo 2015	Detection				X
Renú 2015	Prediction	X	X		
Watanabe 2014	Detection	X	X	X	
Morhard 2013	Detection	X	X		
Shinohara 2013	Technical			X	
Tijssen 2013	Detection	X	X	X	
Phan 2012	Detection	X	X		
Gupta 2010	Detection	X	X		
Zhang 2010	Detection		X		X
Ferda 2009	Detection	X			
Watanabe 2008	Detection				X

Techniques include virtual non-contrast (VNC), iodine maps (IM), virtual monoenergetic images (VMI), and others (e.g., bone removal, X-map)

### Meta-analysis of the diagnostic accuracy of DECT in ICH vs contrast staining

**Fig. 3:**
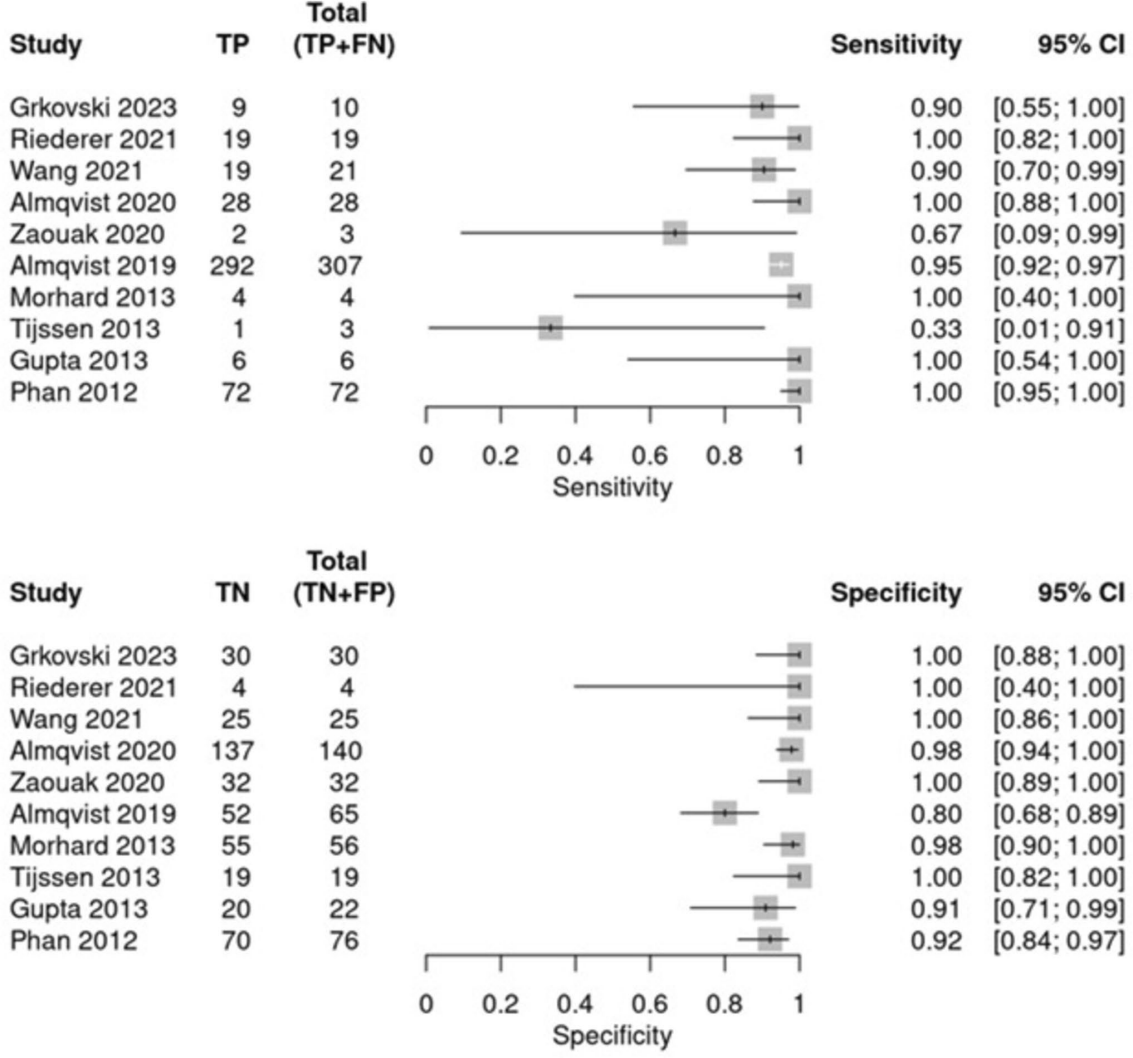
Forest plots summarizing the sensitivity and specificity of the included studies. The top panel depicts sensitivity, and the bottom panel shows specificity. Each study is represented by a square, with the size proportional to its weight in the analysis

**Fig. 4:**
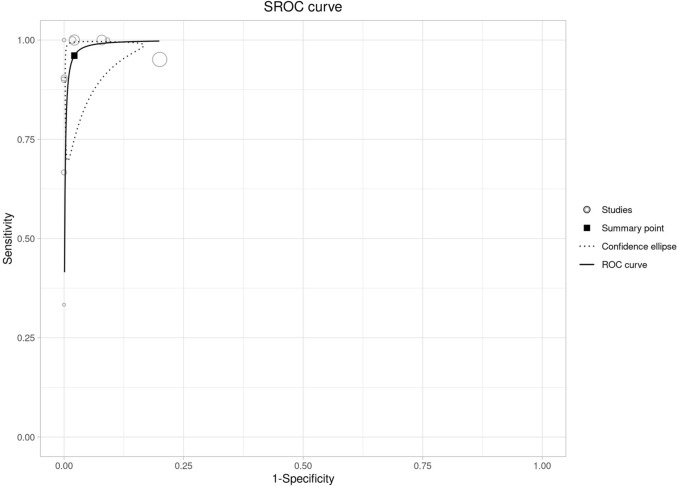
Summary receiver operating characteristic (SROC) curve displaying the diagnostic performance of the included studies. Each circle represents an individual study, with the size proportional to the study’s weight in the analysis. The solid line represents the fitted ROC curve, while the dotted line shows the confidence ellipse around the summary point, indicating the overall sensitivity and specificity

Of the 23 studies assessing the differentiation between contrast staining and ICH, only 10 provided complete diagnostic accuracy data and were included in the meta-analysis. The remaining studies, which lacked extractable contingency data or focused on technical and qualitative outcomes, were analyzed descriptively. For sensitivity, the pooled estimate was 96.1% (95% CI 83.8%–99.1%), with heterogeneity equal to *I*^2^ = 24.5% (Fig. 3A). For specificity, the pooled estimate was 97.8% (95% CI 91.4%–99.5%), with heterogeneity equal to *I*^2^ = 50.5% (Fig. 3B). The sROC analysis yielded an AUC of 0.98 (95% CI 0.96–0.99), confirming the excellent diagnostic accuracy of DECT for differentiating ICH from contrast staining, as shown in Fig. 4.

### Meta-analysis of DECT radiation dose

**Fig. 5:**
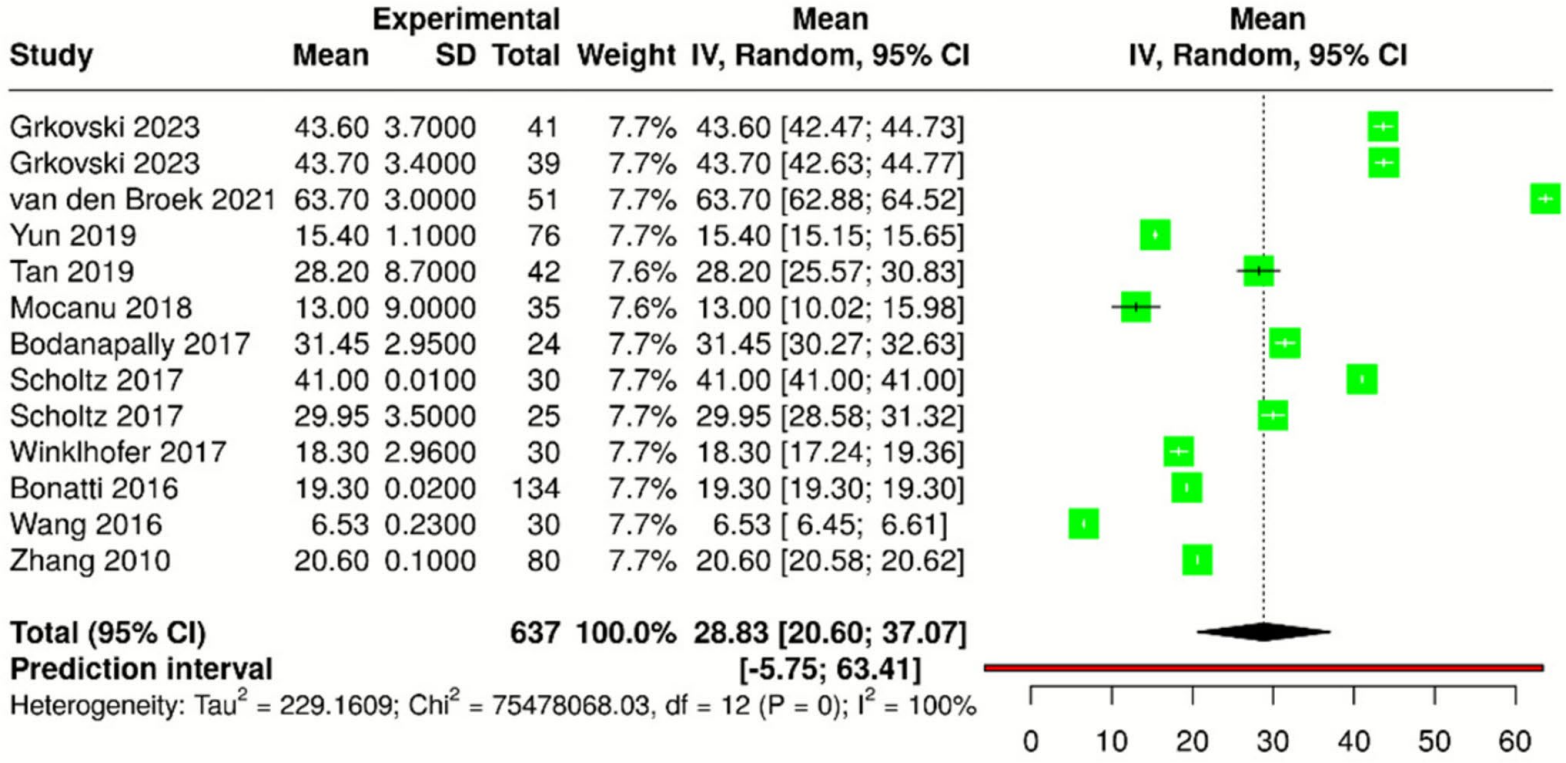
Forest plot showing the mean differences in CT dose index volume (CTDI*vol*) across multiple studies in a meta-analysis. Each study is represented by a green square, with the size proportional to its weight in the analysis. Horizontal lines indicate the 95% confidence intervals (CIs). The diamond at the bottom represents the overall pooled estimate with its 95% CI

**Fig. 6:**
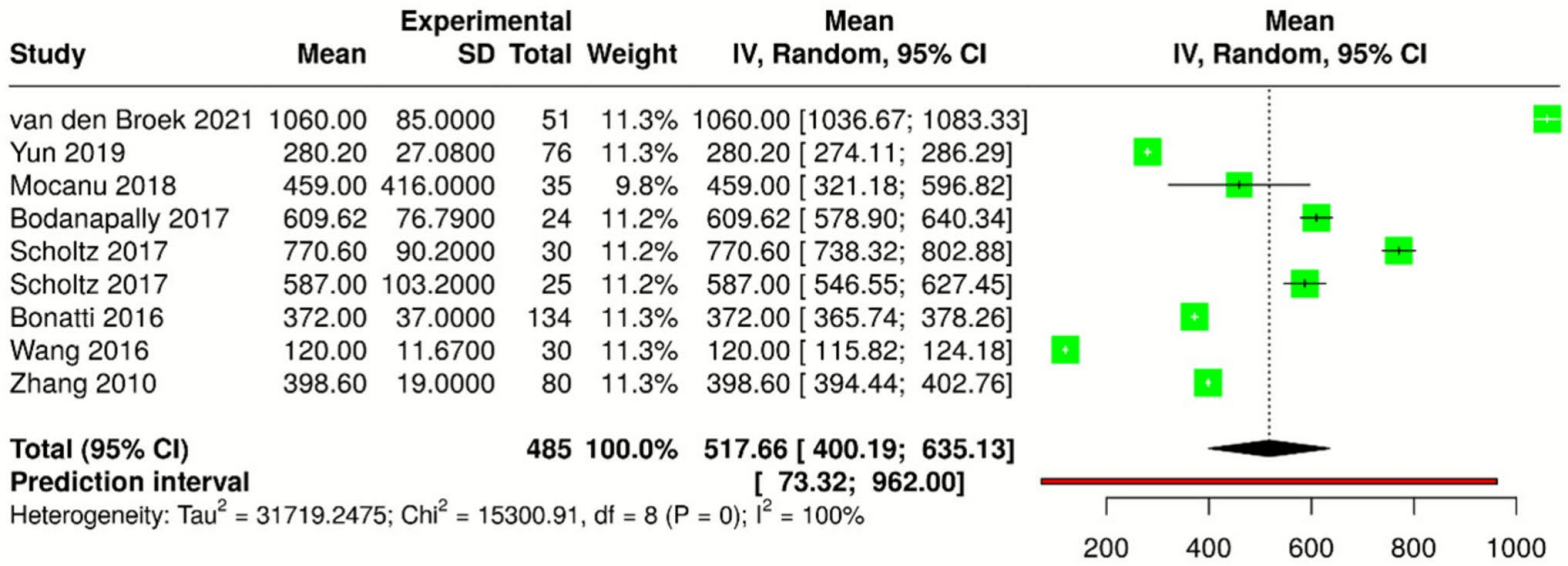
Forest plot showing the mean differences in dose-length product (DLP) across multiple studies in a meta-analysis. Each study is represented by a green square, with the size proportional to its weight in the analysis. Horizontal lines indicate the 95% confidence intervals (CIs). The diamond at the bottom represents the overall pooled estimate with its 95% CI

Data on CTDIvol were reported in 31 out of 68 articles (46%), with values ranging from 6 to 80 mGy. Data on DLP were available in 11 out of 68 articles (16%), with reported values ranging from 120 to 770.6 mGy × cm. Details of the radiation dose data are summarized in Table 2. A meta-analysis of CTDIvol and DLP was conducted only for articles that reported both the median and standard deviation, using a random-effects model with the inverse variance method to account for variability across studies. For CTDIvol (Fig. 5), data were extracted from 13 study parts comprising a total of 637 subjects. The pooled estimate was 28.83 mGy (95% CI 20.60–37.07 mGy). For DLP (Fig. 6), data were analyzed from 9 study parts with a total of 485 subjects, resulting in a pooled estimate of 517.66 mGy × cm (95% CI 400.19–635.13 mGy × cm). Significant heterogeneity was observed for both CTDIvol and DLP (*I*^2^ = 100%, *p* < 0.01), indicating substantial variability in effect sizes across studies in terms of magnitude and/or direction.

### Quality assessment

The QualSyst results, presented in Table 4, indicate that most articles achieved scores ranging from 14 (the minimum value), obtained by Noguchi et al. [67], to 20. Fourteen articles received the highest possible score of 20 points.

**Table 4:** Quality assessment of studies using the QualSyst tool (11), based on the checklist for qualitative studies

Study ID	Question/objective sufficiently described?	Study design evident and appropriate?	Context for the study clear?	Connection to a theoretical framework/wider body of knowledge?	Sampling strategy described, relevant, and justified?	Data collection methods clearly described and systematic?	Data analysis clearly described and systematic?	Use of verification procedure(s) to establish credibility?	Conclusions supported by the results?	Reflexivity of the account?	Total
Robbe 2024	2	2	2	2	2	2	2	2	2	2	20
Cui 2024	2	2	2	2	1	2	2	0	2	2	17
Dai 2023	2	2	2	2	1	2	2	2	2	2	19
Pinckaers 2023	2	2	2	2	2	2	2	2	2	2	20
Grkovski 2023	2	2	2	2	1	2	2	1	2	2	18
Jiang 2023	2	2	2	2	1	2	2	1	2	1	17
Coorens 2023	2	2	2	2	1	2	2	1	2	2	18
Huijberts 2023	2	2	2	2	2	2	2	2	2	2	20
Grkovski 2022	2	2	2	2	1	2	2	2	2	2	19
Ma 2022	2	2	2	2	2	2	2	1	2	2	19
Kauw 2022	2	2	2	2	2	2	2	2	2	2	20
Dodig 2022	2	2	2	2	1	2	2	1	2	2	18
Renu 2022	2	2	2	2	1	2	2	1	2	2	18
Chrzan 2022	2	2	2	2	1	2	2	1	2	2	18
van Ommen 2021	2	2	2	1	1	2	2	1	2	2	17
van den Broek 2021	2	2	2	2	1	2	2	1	2	2	18
Chen 2021	2	2	2	2	1	1	2	2	2	2	18
Fransson 2021	2	2	2	1	0	2	2	1	2	1	16
Wang 2021	2	2	2	2	1	2	2	2	2	2	19
Wolman 2021	2	2	2	2	1	2	2	2	2	2	19
Riederer 2021	2	2	2	2	1	2	1	1	2	2	17
Liu 2021	2	2	2	1	2	2	2	1	2	2	18
van Ommen 2020	2	2	2	2	1	2	2	1	2	2	18
Cai 2020	2	2	2	1	1	2	1	1	2	2	16
Ma 2020	2	2	2	2	1	2	2	2	2	2	19
Liu 2020	2	2	2	2	2	2	2	1	2	2	19
Almqvist 2020	2	2	2	1	1	1	2	1	2	2	16
Wiggins 2020	2	2	2	2	2	2	2	2	2	2	20
Byrne 2020	2	2	2	2	1	2	2	2	2	2	19
Ståhl 2020	2	2	2	2	1	2	2	1	2	2	18
Zaouak 2020	2	2	2	2	1	1	2	1	2	2	17
Zaouak 2019	2	2	2	2	2	2	2	2	2	2	20
Almqvist 2019	2	2	2	2	1	1	1	1	2	2	16
Ebashi 2019	2	2	2	2	2	2	2	2	2	2	20
Yun 2019	2	2	2	2	2	1	1	1	1	2	16
Bodanapally 2019	2	2	2	2	1	1	1	2	1	2	16
Tan 2019	2	2	2	2	2	2	2	1	2	2	19
An 2019	2	2	2	2	2	2	2	2	1	2	19
Eduardo Murias	2	2	2	2	2	2	1	2	1	2	18
Mocanu 2018	2	2	2	2	1	1	1	1	1	2	15
Grams 2018	2	2	2	2	2	2	2	2	2	2	20
Taguchi 2018	2	2	2	2	1	1	2	2	2	2	18
Bodanapally 2018	2	2	2	2	1	1	2	2	2	2	18
Bonatti 2018	2	2	2	2	2	2	2	2	1	2	19
Naruto 2018	2	2	2	2	1	1	1	1	1	2	15
Leithner 2017	2	2	2	2	2	1	1	1	2	2	17
Bodanapally 2017	2	2	2	2	2	2	2	2	2	2	20
Scholtz 2017	2	2	2	2	2	2	2	2	2	2	20
Winklhofer 2017	2	2	2	2	1	1	1	2	2	2	17
Bonatti 2016	2	2	2	2	2	2	2	1	2	2	19
Djurdjevic 2016	2	2	2	2	2	2	2	2	1	2	19
Noguchi 2016	2	2	2	1	1	1	1	1	1	2	14
Yang 2016	2	2	2	2	1	1	1	1	2	2	16
Wang 2016	2	2	2	2	2	2	2	2	2	2	20
Hixson 2016	2	2	2	2	1	1	1	1	1	2	15
Gariani 2015	2	2	2	2	2	1	1	1	2	2	17
Guo 2015	2	2	2	2	1	1	1	1	1	2	15
Renú 2015	2	2	2	2	2	2	2	2	2	2	20
Grams 2015	2	2	2	2	2	2	1	2	1	2	18
Watanabe 2014	2	2	2	2	2	2	1	1	1	2	17
Tijssen 2013	2	2	2	2	1	2	2	1	2	1	17
Morhard 2013	2	2	2	2	2	2	2	2	2	2	20
Shinohara 2013	2	2	2	2	1	1	1	1	1	2	15
Phan 2012	2	2	2	1	1	1	2	2	2	1	16
Zhang 2010	2	2	2	2	2	1	1	1	2	2	17
Gupta 2010	2	2	2	2	2	2	2	2	2	2	20
Ferda 2009	2	2	2	2	1	2	1	1	2	2	17
Watanabe 2008	2	2	2	2	2	1	1	2	2	2	18

The QUADAS-2 tool, on the other hand, indicated a low probability of risk of bias for the data, including in terms of applicability. The quality results are presented in Table 5.

**Table 5:**
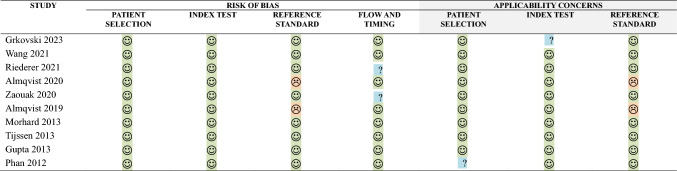
Results from the Quality Assessment of Diagnostic Accuracy Studies (QUADAS-2) (12) criteria for the evaluation of studies included in the systematic review and meta-analysis

Low Risk High Risk Unclear Risk

## Discussion

This systematic review and meta-analysis, based on an analysis of 68 studies published over the past 16 years, analyzed diagnostic and technical advantages of DECT, its limitations, and its comparative role with conventional SECT. One of the most noteworthy outcomes is DECT’s diagnostic accuracy in distinguishing ICH from contrast staining, with a pooled sensitivity and specificity of 96.1% and 97.8%, respectively. This is a substantial improvement over SECT, which often struggles with this differentiation due to overlapping attenuation values [89].

Ebaid et al. [90] reported pooled sensitivity and specificity for detecting ICH as 69.9% and 100%, respectively. Our sub-analysis, specifically focused on differentiating ICH from contrast staining, revealed a much higher diagnostic accuracy. This narrowed focus aligns more closely with DECT’s optimal clinical utility, as distinguishing ICH from contrast staining is critical for therapeutic management in the post-endovascular treatment setting.

In terms of DECT radiation doses, the pooled estimates for CTDIvol (28.83 mGy) and DLP (517.66 mGy × cm) from this meta-analysis are comparable to those reported in SECT studies, suggesting that DECT does not increase radiation exposure [91]. However, the high heterogeneity observed for DLP (*I*^2^ = 99.98%) indicates significant variability in dose parameters across studies, potentially influenced by differences in patient populations, scanner types, and clinical protocols such as the distinction between contrast-enhanced DECT protocols (DECT angiography or perfusion) and non-contrast DECT. While this inclusive approach allowed us to capture real-world variability, protocol-specific analyses would likely yield narrower confidence intervals and more homogeneous estimates. However, such stratified pooling was beyond the primary objective of this review, which aimed to provide a global overview of radiation exposure in cerebrovascular DECT. Indeed, a non-significant trend toward dose reduction over time was identified. This trend aligns with technological advancements and ongoing efforts to optimize imaging protocols, particularly in vulnerable populations such as pediatric and elderly patients [92].

Given the comparable radiation dose and the significant diagnostic accuracy in differentiating contrast staining from ICH, DECT should be the preferred modality in these specific clinical scenarios. Its superior diagnostic performance provides critical value in post-treatment settings, where precise differentiation between contrast staining and ICH can directly impact patient management and outcomes. Beyond the specific differentiation between ICH and contrast staining, several studies included in our review also investigated additional cerebrovascular applications of DECT. These encompassed the detection of early ischemic parenchymal changes through virtual non-contrast or virtual monoenergetic reconstructions, the evaluation of blood–brain barrier integrity by quantifying iodine extravasation as a marker of disruption and the prediction of subsequent hemorrhagic transformation after endovascular therapy or thrombolysis. Although not the primary focus of our analysis, these applications suggest that DECT may provide useful supplementary information in acute stroke care, complementing its established role in differentiating hemorrhage from contrast staining.

Among the studies included, dual-source DECT was the most used technology, reported by 85% of articles, followed by fast kVp-switching (9%) and spectral detectors (3%). The most common acquisition parameters involved a low-energy kVp of 80 and a high-energy kVp of 140, utilized in 50% of studies. This combination is likely to provide an optimal balance between diagnostic performance, material differentiation, and radiation dose.

This review revealed that DECT studies in stroke were predominantly focused in three clinical scenarios: stroke detection (50%), outcome prediction in stroke (24%), and technical CT aspects (26%). These applications demonstrate DECT’s versatility in addressing both diagnostic and prognostic challenges in stroke imaging. For instance, Byrne et al. [49] showed that the parenchymal relative iodine concentration at DECT compared to the superior sagittal sinus can reliably predict ICH development, serving as a useful imaging biomarker for risk stratification after endovascular treatment. Additionally, early grading of blood–brain barrier disruption has been shown to be useful to identify patients at increased risk of delayed hemorrhagic transformation [77, 93].

Despite its numerous advantages, DECT faces several challenges that must be addressed to fully realize its potential.

### Limitations

This review has certain limitations. First, some of the studies included may have shared or duplicated patient data, particularly among studies from the same research group. This overlap can impact the quality of the review [94, 95]. Preventing this issue would require access to individual patient data across studies [96]. Second, many studies underreported key technical details, such as contrast agent type, concentration, and flow rate. These parameters can significantly influence image quality and diagnostic performance, underscoring the need for more comprehensive reporting standards in future research. Third, significant heterogeneity was observed in radiation dose estimates across studies, which reflects differences in protocols, scanner technologies, and patient populations. Although a random-effects model was used to account for between-study variability, the limited number and heterogeneity of available studies did not allow for stable estimation of variance components (*τ*^2^) or prediction regions on the SROC. Fourth, the relatively low adoption of advanced DECT technologies limits the applicability of the results to centers equipped with dual-source systems. Lastly, while this review includes a substantial number of studies, it is mainly retrospective (55/68, 81%), with only 13/68 (19%) prospective, which may introduce biases.

Standardization of acquisition protocols and post-processing techniques will be crucial for reducing variability and improving diagnostic accuracy. Additionally, exploring DECT’s potential in other neurovascular conditions, such as chronic cerebrovascular diseases and TIAs, could broaden its clinical utility.

## Conclusion

This systematic review underscores the potential of DECT in stroke imaging, offering superior diagnostic performance in detecting ICH versus contrast staining after endovascular therapies, comparable radiation doses to SECT, and innovative post-processing capabilities. The large variability in voltage and dose among different protocols reflects the relative immaturity of DECT and the need of multicentric harmonization and standardization. Given its high diagnostic accuracy and comparable radiation exposure to SECT, where technically available, DECT should always be considered in the specific scenario of differentiating ICH from contrast staining.

This systematic review sets the foundation for potential applications in Photon Counting CT, further expanding its diagnostic capabilities in cerebrovascular imaging.

## Supplementary Information

Below is the link to the electronic supplementary material.

Supplementary file1 (DOCX 15 KB)

## References

[1] Lindsay MP, Norrving B, Sacco RL et al (2019) World Stroke Organization (WSO): global stroke fact sheet 2019. Int J Stroke 14:806–817. 10.1177/1747493019881353 31658892 10.1177/1747493019881353

[2] Lin MP, Liebeskind DS (2016) Imaging of ischemic stroke. Continuum 22:1399–1423. 10.1212/CON.0000000000000376 27740982 10.1212/CON.0000000000000376 PMC5898964

[3] Srinivasan A, Goyal M, Azri FA, Lum C (2006) State-of-the-art imaging of acute stroke. Radiographics 26:S75–S95. 10.1148/rg.26si065501 17050521 10.1148/rg.26si065501

[4] Caldwell J, Heran MKS, McGuinness B, Barber PA (2017) Imaging in acute ischaemic stroke: pearls and pitfalls. Pract Neurol 17:349–358. 10.1136/practneurol-2016-001569 28743791 10.1136/practneurol-2016-001569

[5] Jadhav AP, Desai SM, Liebeskind DS, Wechsler LR (2020) Neuroimaging of acute stroke. Neurol Clin 38:185–199. 10.1016/j.ncl.2019.09.004 31761058 10.1016/j.ncl.2019.09.004

[6] Chen W, Wu J, Wei R et al (2022) Improving the diagnosis of acute ischemic stroke on non-contrast CT using deep learning: a multicenter study. Insights Imaging 13:184. 10.1186/s13244-022-01331-3 36471022 10.1186/s13244-022-01331-3 PMC9723089

[7] Chen S, Zhang J, Quan X et al (2022) Diagnostic accuracy of dual-energy computed tomography to differentiate intracerebral hemorrhage from contrast extravasation after endovascular thrombectomy for acute ischemic stroke: systematic review and meta-analysis. Eur Radiol 32:432–441. 10.1007/s00330-021-08212-1 34327578 10.1007/s00330-021-08212-1

[8] Ebashi R, Ogata A, Nishihara M et al (2019) Significance of simulated conventional images on dual energy CT after endovascular treatment for ischemic stroke. J Neurointerv Surg 11:898–902. 10.1136/neurintsurg-2018-014486 30670626 10.1136/neurintsurg-2018-014486

[9] Moher D, Liberati A, Tetzlaff J, Altman DG (2009) Preferred reporting items for systematic reviews and meta-analyses: the PRISMA statement. PLoS Med 6:e1000097. 10.1371/journal.pmed.1000097 19621072 10.1371/journal.pmed.1000097 PMC2707599

[10] McInnes MDF, Moher D, Thombs BD et al (2018) Preferred reporting items for a systematic review and meta-analysis of diagnostic test accuracy studies. JAMA 319:388. 10.1001/jama.2017.19163 29362800 10.1001/jama.2017.19163

[11] Kmet LM, Cook LS, Lee RC (2004) Standard quality assessment criteria for evaluating primary research papers from a variety of fields. HTA Initiative # 13

[12] Whiting PF (2011) QUADAS-2: a revised tool for the quality assessment of diagnostic accuracy studies. Ann Intern Med 155:529. 10.7326/0003-4819-155-8-201110180-00009 22007046 10.7326/0003-4819-155-8-201110180-00009

[13] Chu H, Cole SR (2006) Bivariate meta-analysis of sensitivity and specificity with sparse data: a generalized linear mixed model approach. J Clin Epidemiol 59:1331–1332. 10.1016/j.jclinepi.2006.06.011 17098577 10.1016/j.jclinepi.2006.06.011

[14] Bates D, Mächler M, Bolker B, Walker S (2015) Fitting linear mixed-effects models using lme4. J Stat Softw. 10.18637/jss.v067.i01

[15] Plana MN, Pérez T, Zamora J (2021) New measures improved the reporting of heterogeneity in diagnostic test accuracy reviews: a metaepidemiological study. J Clin Epidemiol 131:101–112. 10.1016/j.jclinepi.2020.11.011 33227449 10.1016/j.jclinepi.2020.11.011

[16] Reitsma JB, Glas AS, Rutjes AWS et al (2005) Bivariate analysis of sensitivity and specificity produces informative summary measures in diagnostic reviews. J Clin Epidemiol 58:982–990. 10.1016/j.jclinepi.2005.02.022 16168343 10.1016/j.jclinepi.2005.02.022

[17] Zamora J, Abraira V, Muriel A et al (2006) Meta-DiSc: a software for meta-analysis of test accuracy data. BMC Med Res Methodol 6:31. 10.1186/1471-2288-6-31 16836745 10.1186/1471-2288-6-31 PMC1552081

[18] Zhou Y, Dendukuri N (2014) Statistics for quantifying heterogeneity in univariate and bivariate meta-analyses of binary data: the case of meta-analyses of diagnostic accuracy. Stat Med 33:2701–2717. 10.1002/sim.6115 24903142 10.1002/sim.6115

[19] DerSimonian R, Laird N (2015) Meta-analysis in clinical trials revisited. Contemp Clin Trials 45:139–145. 10.1016/j.cct.2015.09.002 26343745 10.1016/j.cct.2015.09.002 PMC4639420

[20] Migliavaca CB, Stein C, Colpani V et al (2022) Meta-analysis of prevalence: <scp> *I*^2^ </scp> statistic and how to deal with heterogeneity. Res Synth Methods 13:363–367. 10.1002/jrsm.1547 35088937 10.1002/jrsm.1547

[21] Shim SR, Kim S-J, Lee J (2019) Diagnostic test accuracy: application and practice using R software. Epidemiol Health 41:e2019007. 10.4178/epih.e2019007 30999739 10.4178/epih.e2019007 PMC6545496

[22] Cui Y, Gong W, Duan S et al (2024) Diagnostic value of X-Map images reconstructed by plain dual-energy computed tomography scans in acute ischemic stroke. Curr Med Imaging Rev. 10.2174/0115734056294190240522113039 10.2174/0115734056294190240522113039 38803185

[23] Robbe MMQ, Pinckaers FME, van Oostenbrugge RJ et al (2024) The correlation between CT perfusion deficits and immediate post-endovascular treatment contrast extravasation on dual energy CT in acute ischemic stroke patients. Eur J Radiol 173:111379. 10.1016/j.ejrad.2024.111379 38387339 10.1016/j.ejrad.2024.111379

[24] Pinckaers FM, Mentink MM, Boogaarts HD et al (2023) Early post-endovascular treatment contrast extravasation on dual-energy CT is associated with clinical and radiological stroke outcomes: a 10-year single-centre experience. Eur Stroke J 8:508–516. 10.1177/23969873231157901 37231689 10.1177/23969873231157901 PMC10334176

[25] Jiang J, Gu H, Li M et al (2023) The value of dual-energy computed tomography angiography-derived parameters in the evaluation of clot composition. Acad Radiol 30:1866–1873. 10.1016/j.acra.2022.12.023 36587997 10.1016/j.acra.2022.12.023

[26] Coorens NA, Lipman KG, Krishnam SP et al (2023) Intracerebral hemorrhage segmentation on noncontrast computed tomography using a masked loss function U-Net approach. J Comput Assist Tomogr 47:93–101. 10.1097/RCT.0000000000001380 36219722 10.1097/RCT.0000000000001380

[27] Huijberts I, Pinckaers FME, van Zwam WH et al (2023) Cerebral arterial air emboli on immediate post-endovascular treatment CT are associated with poor short- and long-term clinical outcomes in acute ischaemic stroke patients. J Neuroradiol 50:530–536. 10.1016/j.neurad.2023.06.001 37331695 10.1016/j.neurad.2023.06.001

[28] Ma C, Xu D, Hui Q et al (2022) Quantitative intracerebral iodine extravasation in risk stratification for intracranial hemorrhage in patients with acute ischemic stroke. AJNR Am J Neuroradiol 43:1589–1596. 10.3174/ajnr.A7671 36202552 10.3174/ajnr.A7671 PMC9731239

[29] Kauw F, Ding VY, Dankbaar JW et al (2022) Detection of early ischemic changes with virtual noncontrast dual-energy CT in acute ischemic stroke: a noninferiority analysis. AJNR Am J Neuroradiol 43:1259–1264. 10.3174/ajnr.A7600 35953275 10.3174/ajnr.A7600 PMC9451625

[30] Dodig D, Matana Kaštelan Z, Bartolović N et al (2022) Virtual monoenergetic dual-energy CT reconstructions at 80 keV are optimal non-contrast CT technique for early stroke detection. Neuroradiol J 35:337–345. 10.1177/19714009211047449 34550827 10.1177/19714009211047449 PMC9244738

[31] Renú A, Laredo C, Rodríguez-Vázquez A et al (2022) Characterization of subarachnoid hyperdensities after thrombectomy for acute stroke using dual-energy CT. Neurology. 10.1212/WNL.0000000000013198 34921104 10.1212/WNL.0000000000013198

[32] van den Broek M, Byrne D, Lyndon D et al (2022) ASPECTS estimation using dual-energy CTA-derived virtual non-contrast in large vessel occlusion acute ischemic stroke: a dose reduction opportunity for patients undergoing repeat CT? Neuroradiology 64:483–491. 10.1007/s00234-021-02773-0 34379143 10.1007/s00234-021-02773-0

[33] Chen J, Niu Z, Zhan K et al (2021) Evaluation of modified calcium removal algorithm in dual energy CT of internal carotid artery. Eur J Radiol 145:109927. 10.1016/j.ejrad.2021.109927 34773829 10.1016/j.ejrad.2021.109927

[34] Fransson V, Mellander H, Wasselius J, Ydström K (2021) Detection of perfusion deficits in multiphase computed tomography angiography—a stroke imaging technique based on iodine mapping on spectral computed tomography: initial findings. J Comput Assist Tomogr 45:618–624. 10.1097/RCT.0000000000001173 34176878 10.1097/RCT.0000000000001173

[35] Wang Z, Chen W, Lin H et al (2021) Early diagnosis and prediction of intracranial hemorrhage using dual-energy computed tomography after mechanical thrombectomy in patients with acute ischemic stroke. Clin Neurol Neurosurg 203:106551. 10.1016/j.clineuro.2021.106551 33636506 10.1016/j.clineuro.2021.106551

[36] Wolman DN, van Ommen F, Tong E et al (2021) Non-contrast dual-energy CT virtual ischemia maps accurately estimate ischemic core size in large-vessel occlusive stroke. Sci Rep 11:6745. 10.1038/s41598-021-85143-3 33762589 10.1038/s41598-021-85143-3 PMC7991428

[37] Liu K, Jiang L, Zhao Y et al (2021) Risk factors of contrast extravasation and subsequent hemorrhagic transformation after thrombectomy. J Int Med Res. 10.1177/03000605211049074 34633880 10.1177/03000605211049074 PMC8511932

[38] Cai J, Zhou Y, Zhao Y et al (2021) Comparison of various reconstructions derived from dual-energy CT immediately after endovascular treatment of acute ischemic stroke in predicting hemorrhage. Eur Radiol 31:4419–4427. 10.1007/s00330-020-07574-2 33389034 10.1007/s00330-020-07574-2

[39] Ståhl F, Gontu V, Almqvist H et al (2021) Performance of dual layer dual energy CT virtual monoenergetic images to identify early ischemic changes in patients with anterior circulation large vessel occlusion. J Neuroradiol 48:75–81. 10.1016/j.neurad.2020.12.002 33340643 10.1016/j.neurad.2020.12.002

[40] Zaouak Y, Sadeghi N, Sarbu N et al (2020) Differentiation between cerebral hemorrhage and contrast extravasation using dual energy computed tomography after intra-arterial neuro interventional procedures. J Belg Soc Radiol. 10.5334/jbsr.2083 33283150 10.5334/jbsr.2083 PMC7693760

[41] Chrzan R, Łasocha B, Brzegowy P, Popiela T (2022) Dual energy computed tomography in differentiation of iodine contrast agent staining from secondary brain haemorrhage in patients with ischaemic stroke treated with thrombectomy. Neurol Neurochir Pol 56:68–74. 10.5603/PJNNS.a2022.0005 34985116 10.5603/PJNNS.a2022.0005

[42] Grkovski R, Acu L, Ahmadli U et al (2023) Dual-energy computed tomography in stroke imaging. Clin Neuroradiol 33:747–754. 10.1007/s00062-023-01270-6 36862231 10.1007/s00062-023-01270-6 PMC10450017

[43] Dai Y, Xu H, Fang X et al (2023) Dual-energy CT in assessment of thrombus perviousness and its application in predicting outcomes after intravenous thrombolysis in acute ischemic stroke. Eur J Radiol 164:110861. 10.1016/j.ejrad.2023.110861 37167682 10.1016/j.ejrad.2023.110861

[44] van Ommen F, Bennink E, Dankbaar JW et al (2021) Improving the quality of cerebral perfusion maps with monoenergetic dual-energy computed tomography reconstructions. J Comput Assist Tomogr 45:103–109. 10.1097/RCT.0000000000000981 32176156 10.1097/RCT.0000000000000981

[45] Ma C, Hui Q, Gao X et al (2021) The feasibility of dual-energy CT to predict the probability of symptomatic intracerebral haemorrhage after successful mechanical thrombectomy. Clin Radiol 76:316.e9-316.e18. 10.1016/j.crad.2020.12.013 33509606 10.1016/j.crad.2020.12.013

[46] Liu K, Jiang L, Ruan J et al (2020) The role of dual energy CT in evaluating hemorrhagic complications at different stages after thrombectomy. Front Neurol. 10.3389/fneur.2020.583411 33117268 10.3389/fneur.2020.583411 PMC7575741

[47] Almqvist H, Almqvist NS, Holmin S, Mazya MV (2020) Dual-energy CT follow-up after stroke thrombolysis alters assessment of hemorrhagic complications. Front Neurol. 10.3389/fneur.2020.00357 32508735 10.3389/fneur.2020.00357 PMC7249255

[48] Wiggins WF, Potter CA, Sodickson AD (2020) Dual-energy CT to differentiate small foci of intracranial hemorrhage from calcium. Radiology 294:129–138. 10.1148/radiol.2019190792 31687919 10.1148/radiol.2019190792

[49] Byrne D, Walsh JP, Schmiedeskamp H et al (2020) Prediction of hemorrhage after successful recanalization in patients with acute ischemic stroke: improved risk stratification using dual-energy CT parenchymal iodine concentration ratio relative to the superior sagittal sinus. AJNR Am J Neuroradiol 41:64–70. 10.3174/ajnr.A6345 31896566 10.3174/ajnr.A6345 PMC6975329

[50] Almqvist H, Holmin S, Mazya MV (2019) Dual energy CT after stroke thrombectomy alters assessment of hemorrhagic complications. Neurology. 10.1212/WNL.0000000000008093 31409735 10.1212/WNL.0000000000008093

[51] Yun SY, Heo YJ, Jeong HW et al (2019) Dual-energy CT angiography-derived virtual non-contrast images for follow-up of patients with surgically clipped aneurysms: a retrospective study. Neuroradiology 61:747–755. 10.1007/s00234-019-02170-8 30684114 10.1007/s00234-019-02170-8

[52] Bodanapally UK, Shanmuganathan K, Ramaswamy M et al (2019) Iodine-based dual-energy CT of traumatic hemorrhagic contusions: relationship to in-hospital mortality and short-term outcome. Radiology 292:730–738. 10.1148/radiol.2019190078 31361206 10.1148/radiol.2019190078 PMC7705608

[53] Tan CO, Lam S, Kuppens D et al (2019) Spot and diffuse signs: quantitative markers of intracranial hematoma expansion at dual-energy CT. Radiology 290:179–186. 10.1148/radiol.2018180322 30375929 10.1148/radiol.2018180322

[54] An H, Zhao W, Wang J et al (2019) Contrast staining may be associated with intracerebral hemorrhage but not functional outcome in acute ischemic stroke patients treated with endovascular thrombectomy. Aging Dis 10:784. 10.14336/AD.2018.0807 31440384 10.14336/AD.2018.0807 PMC6675522

[55] Mocanu I, Van Wettere M, Absil J et al (2018) Value of dual-energy CT angiography in patients with treated intracranial aneurysms. Neuroradiology 60:1287–1295. 10.1007/s00234-018-2090-5 30219936 10.1007/s00234-018-2090-5

[56] Grams AE, Djurdjevic T, Rehwald R et al (2018) Improved visualisation of early cerebral infarctions after endovascular stroke therapy using dual-energy computed tomography oedema maps. Eur Radiol 28:4534–4541. 10.1007/s00330-018-5449-4 29728814 10.1007/s00330-018-5449-4 PMC6182745

[57] Taguchi K, Itoh T, Fuld MK et al (2018) “X-map 2.0” for edema signal enhancement for acute ischemic stroke using non–contrast-enhanced dual-energy computed tomography. Invest Radiol 53:432–439. 10.1097/RLI.0000000000000461 29543692 10.1097/RLI.0000000000000461

[58] Bodanapally UK, Shanmuganathan K, Issa G et al (2018) Dual-energy CT in hemorrhagic progression of cerebral contusion: overestimation of hematoma volumes on standard 120-kV images and rectification with virtual high-energy monochromatic images after contrast-enhanced whole-body imaging. AJNR Am J Neuroradiol 39:658–662. 10.3174/ajnr.A5558 29439124 10.3174/ajnr.A5558 PMC7410769

[59] Bonatti M, Lombardo F, Zamboni GA et al (2018) Iodine extravasation quantification on dual-energy CT of the brain performed after mechanical thrombectomy for acute ischemic stroke can predict hemorrhagic complications. AJNR Am J Neuroradiol 39:441–447. 10.3174/ajnr.A5513 29348131 10.3174/ajnr.A5513 PMC7655335

[60] Naruto N, Tannai H, Nishikawa K et al (2018) Dual-energy bone removal computed tomography (BRCT): preliminary report of efficacy of acute intracranial hemorrhage detection. Emerg Radiol 25:29–33. 10.1007/s10140-017-1558-7 28932923 10.1007/s10140-017-1558-7

[61] Leithner D, Mahmoudi S, Wichmann JL et al (2018) Evaluation of virtual monoenergetic imaging algorithms for dual-energy carotid and intracerebral CT angiography: effects on image quality, artefacts and diagnostic performance for the detection of stenosis. Eur J Radiol 99:111–117. 10.1016/j.ejrad.2017.12.024 29362140 10.1016/j.ejrad.2017.12.024

[62] Bodanapally UK, Dreizin D, Issa G et al (2017) Dual-energy CT in enhancing subdural effusions that masquerade as subdural hematomas: diagnosis with virtual high-monochromatic (190-keV) images. AJNR Am J Neuroradiol 38:1946–1952. 10.3174/ajnr.A5318 28798216 10.3174/ajnr.A5318 PMC7963601

[63] Scholtz J-E, Wichmann JL, Bennett DW et al (2017) Detecting intracranial hemorrhage using automatic tube current modulation with advanced modeled iterative reconstruction in unenhanced head single- and dual-energy dual-source CT. AJR Am J Roentgenol 208:1089–1096. 10.2214/AJR.16.17171 28245141 10.2214/AJR.16.17171

[64] Winklhofer S, Vittoria De Martini I, Nern C et al (2017) Dual-energy computed tomography in stroke imaging: technical and clinical considerations of virtual noncontrast images for detection of the hyperdense artery sign. J Comput Assist Tomogr 41:843–848. 10.1097/RCT.0000000000000638 28708725 10.1097/RCT.0000000000000638

[65] Bonatti M, Lombardo F, Zamboni GA et al (2017) Dual-energy CT of the brain: Comparison between DECT angiography-derived virtual unenhanced images and true unenhanced images in the detection of intracranial haemorrhage. Eur Radiol 27:2690–2697. 10.1007/s00330-016-4658-y 27882426 10.1007/s00330-016-4658-y

[66] Djurdjevic T, Rehwald R, Knoflach M et al (2017) Prediction of infarction development after endovascular stroke therapy with dual-energy computed tomography. Eur Radiol 27:907–917. 10.1007/s00330-016-4412-5 27255400 10.1007/s00330-016-4412-5 PMC5591619

[67] Noguchi K, Itoh T, Naruto N et al (2017) A novel imaging technique (X-Map) to identify acute ischemic lesions using noncontrast dual-energy computed tomography. J Stroke Cerebrovasc Dis 26:34–41. 10.1016/j.jstrokecerebrovasdis.2016.08.025 27639587 10.1016/j.jstrokecerebrovasdis.2016.08.025

[68] Yang B, Gao Y, Yang Y-Y, Zhao W (2016) Application value of selective photon shield in dual-energy computed tomography angiography for diagnosis of intracranial aneurysms. J Craniofac Surg 27:e265–e270. 10.1097/SCS.0000000000002496 27035604 10.1097/SCS.0000000000002496

[69] Wang D, Zhang Q, Hu H et al (2016) Optimal contrast of cerebral dual-energy computed tomography angiography in patients with spontaneous subarachnoid hemorrhage. J Comput Assist Tomogr 40:48–52. 10.1097/RCT.0000000000000336 26571057 10.1097/RCT.0000000000000336 PMC4718178

[70] Hixson HR, Leiva-Salinas C, Sumer S et al (2016) Utilizing dual energy CT to improve CT diagnosis of posterior fossa ischemia. J Neuroradiol 43:346–352. 10.1016/j.neurad.2016.04.001 27255679 10.1016/j.neurad.2016.04.001

[71] Gariani J, Cuvinciuc V, Courvoisier D et al (2016) Diagnosis of acute ischemia using dual energy CT after mechanical thrombectomy. J Neurointerv Surg 8:996–1000. 10.1136/neurintsurg-2015-011988 26534867 10.1136/neurintsurg-2015-011988

[72] Guo Y, Ou S-X, Qian M et al (2015) Dual-energy CT angiography for the diagnosis of intracranial dural arteriovenous fistula. Int J Clin Exp Med 8:7802–7808 26221332 PMC4509277

[73] Renú A, Amaro S, Laredo C et al (2015) Relevance of blood–brain barrier disruption after endovascular treatment of ischemic stroke. Stroke 46:673–679. 10.1161/STROKEAHA.114.008147 25657188 10.1161/STROKEAHA.114.008147

[74] Grams AE, Knoflach M, Rehwald R et al (2015) Residual thromboembolic material in cerebral arteries after endovascular stroke therapy can be identified by dual-energy CT. AJNR Am J Neuroradiol 36:1413–1418. 10.3174/ajnr.A4350 25999414 10.3174/ajnr.A4350 PMC7964684

[75] Watanabe Y, Tsukabe A, Kunitomi Y et al (2014) Dual-energy CT for detection of contrast enhancement or leakage within high-density haematomas in patients with intracranial haemorrhage. Neuroradiology 56:291–295. 10.1007/s00234-014-1333-3 24510167 10.1007/s00234-014-1333-3

[76] Grkovski R, Acu L, Ahmadli U et al (2023) A novel dual-energy CT method for detection and differentiation of intracerebral hemorrhage from contrast extravasation in stroke patients after endovascular thrombectomy. Clin Neuroradiol 33:171–177. 10.1007/s00062-022-01198-3 35960327 10.1007/s00062-022-01198-3 PMC10014653

[77] Morhard D, Ertl L, Gerdsmeier-Petz W et al (2014) Dual-energy CT immediately after endovascular stroke intervention: prognostic implications. Cardiovasc Intervent Radiol 37:1171–1178. 10.1007/s00270-013-0804-y 24310826 10.1007/s00270-013-0804-y

[78] Shinohara Y, Sakamoto M, Iwata N et al (2014) Usefulness of monochromatic imaging with metal artifact reduction software for computed tomography angiography after intracranial aneurysm coil embolization. Acta radiol 55:1015–1023. 10.1177/0284185113510492 24215905 10.1177/0284185113510492

[79] Zhang L-J, Wu S-Y, Poon CS et al (2010) Automatic bone removal dual-energy CT angiography for the evaluation of intracranial aneurysms. J Comput Assist Tomogr 34:816–824. 10.1097/RCT.0b013e3181eff93c 21084894 10.1097/RCT.0b013e3181eff93c

[80] Gupta R, Phan CM, Leidecker C et al (2010) Evaluation of dual-energy CT for differentiating intracerebral hemorrhage from iodinated contrast material staining. Radiology 257:205–211. 10.1148/radiol.10091806 20679449 10.1148/radiol.10091806

[81] Ferda J, Novák M, Mírka H et al (2009) The assessment of intracranial bleeding with virtual unenhanced imaging by means of dual-energy CT angiography. Eur Radiol 19:2518–2522. 10.1007/s00330-009-1495-2 19585123 10.1007/s00330-009-1495-2

[82] Watanabe Y, Uotani K, Nakazawa T et al (2009) Dual-energy direct bone removal CT angiography for evaluation of intracranial aneurysm or stenosis: comparison with conventional digital subtraction angiography. Eur Radiol 19:1019–1024. 10.1007/s00330-008-1213-5 19002466 10.1007/s00330-008-1213-5

[83] Murias E, Vega P, Lopez-Cancio E et al (2020) Dual energy CT in the management of antiplatelet therapy in patients with acute ischemic stroke for carotid obstruction. Interv Neuroradiol 26:222–230. 10.1177/1591019919880425 31684785 10.1177/1591019919880425 PMC7507223

[84] Phan CM, Yoo AJ, Hirsch JA et al (2012) Differentiation of hemorrhage from iodinated contrast in different intracranial compartments using dual-energy head CT. AJNR Am J Neuroradiol 33:1088–1094. 10.3174/ajnr.A2909 22268092 10.3174/ajnr.A2909 PMC8013231

[85] Riederer I, Fingerle AA, Zimmer C et al (2021) Potential of dual-layer spectral CT for the differentiation between hemorrhage and iodinated contrast medium in the brain after endovascular treatment of ischemic stroke patients. Clin Imaging 79:158–164. 10.1016/j.clinimag.2021.04.020 33962188 10.1016/j.clinimag.2021.04.020

[86] van Ommen F, Dankbaar JW, Zhu G et al (2021) Virtual monochromatic dual-energy CT reconstructions improve detection of cerebral infarct in patients with suspicion of stroke. Neuroradiology 63:41–49. 10.1007/s00234-020-02492-y 32728777 10.1007/s00234-020-02492-y PMC7803871

[87] Zaouak Y, Sadeghi N, Sarbu N et al (2020) Differentiation between cerebral hemorrhage and contrast extravasation using dual energy computed tomography after intra-arterial neuro interventional procedures. J Belgian Soc Radiol. 10.5334/jbsr.2083 10.5334/jbsr.2083 PMC7693760 33283150

[88] Tijssen MPM, Hofman PAM, Stadler AAR et al (2014) The role of dual energy CT in differentiating between brain haemorrhage and contrast medium after mechanical revascularisation in acute ischaemic stroke. Eur Radiol 24:834–840. 10.1007/s00330-013-3073-x 24258277 10.1007/s00330-013-3073-x

[89] Greer DM, Koroshetz WJ, Cullen S et al (2004) Magnetic resonance imaging improves detection of intracerebral hemorrhage over computed tomography after intra-arterial thrombolysis. Stroke 35:491–495. 10.1161/01.STR.0000114201.11353.C5 14739411 10.1161/01.STR.0000114201.11353.C5

[90] Ebaid NY, Mouffokes A, Yasen NS et al (2024) Diagnostic accuracy of dual-energy computed tomography in the diagnosis of neurological complications after endovascular treatment of acute ischaemic stroke: a systematic review and meta-analysis. Br J Radiol 97:73–92. 10.1093/bjr/tqad007 38263833 10.1093/bjr/tqad007 PMC11027317

[91] Tan WS, Foley S, Ryan ML (2023) Investigating CT head diagnostic reference levels based on indication-based protocols—a single site study. Radiography 29:786–791. 10.1016/j.radi.2023.05.003 37267841 10.1016/j.radi.2023.05.003

[92] Dudhe SS, Mishra G, Parihar P et al (2024) Radiation dose optimization in radiology: a comprehensive review of safeguarding patients and preserving image fidelity. Cureus. 10.7759/cureus.60846 38910606 10.7759/cureus.60846 PMC11191847

[93] Arba F, Rinaldi C, Caimano D et al (2021) Blood–brain barrier disruption and hemorrhagic transformation in acute ischemic stroke: systematic review and meta-analysis. Front Neurol. 10.3389/fneur.2020.594613 33551955 10.3389/fneur.2020.594613 PMC7859439

[94] Huston P, Moher D (1996) Redundancy, disaggregation, and the integrity of medical research. Lancet 347:1024–1026. 10.1016/S0140-6736(96)90153-1 8606568 10.1016/s0140-6736(96)90153-1

[95] Murphy L, Wyllie A (2009) Duplicate patient data in a meta-analysis; a threat to validity. J Crit Care 24:466–467. 10.1016/j.jcrc.2008.12.012 19327334 10.1016/j.jcrc.2008.12.012

[96] Sardanelli F, Alì M, Hunink MG et al (2018) To share or not to share? Expected pros and cons of data sharing in radiological research. Eur Radiol 28:2328–2335. 10.1007/s00330-017-5165-5 29349697 10.1007/s00330-017-5165-5

