# Supplementary material for: Diagnostic accuracy of dual-energy CT in distinguishing intracerebral hemorrhage from contrast staining: a systematic review and meta-analysis with radiation dose assessment
Source: Radiol Med. 2026 Feb 11;131(5):772–96. doi: 10.1007/s11547-026-02180-6 (PMC13201350; doi:10.1007/s11547-026-02180-6)
Supplement: Supplementary file 1 — Supplementary file1 (DOCX 15 KB) [file 11547_2026_2180_MOESM1_ESM.docx]

**Supplementary Material**

**Diagnostic accuracy of dual-energy CT for intracerebral hemorrhage vs. contrast staining, radiation dose, and stroke applications: a systematic review and meta-analysis**

Systematic search was conducted on MEDLINE (PubMed) and EMBASE (Elsevier), for studies reporting the use of DECT for clinical applications of brain.

In patients with acute ischemic or hemorrhagic stroke who have undergone endovascular therapy (Population), dual-energy computed tomography (DECT) (Intervention) is employed to differentiate intracerebral hemorrhage (ICH) from contrast staining (Outcome), a critical distinction in post-treatment assessment. While traditional single-energy CT (SECT) or MRI may be used as comparators in some contexts (Comparison), DECT provides advanced post-processing capabilities such as iodine maps and virtual non-contrast images, offering superior diagnostic accuracy with comparable radiation doses.

The search query for MEDLINE (PubMed) was

("dual energy computed tomography"[MeSH Terms] OR "dual energy computed tomography"[Title/Abstract] OR "dual energy CT"[Title/Abstract])

AND

("perfusion"[MeSH Terms] OR "hypoperfusion"[Title/Abstract] OR "microperfusion"[Title/Abstract] OR "perfusion"[Title/Abstract] OR "perfusion theory"[Title/Abstract])

AND

("brain"[MeSH Terms] OR "brain"[Title/Abstract] OR "cerebrum"[Title/Abstract] OR "encephalon"[Title/Abstract] OR "stroke"[MeSH Terms] OR "stroke"[Title/Abstract] OR "cerebrovascular accident"[Title/Abstract] OR "CVA"[Title/Abstract] OR "acute stroke"[Title/Abstract] OR "ischemic stroke"[Title/Abstract] OR "brain ischemic attack"[Title/Abstract] OR "brain vascular accident"[Title/Abstract] OR "cerebral stroke"[Title/Abstract] OR "cryptogenic stroke"[Title/Abstract] OR "ischemic seizure"[Title/Abstract])

AND

(english[lang])

AND

("journal article"[Publication Type] OR "review"[Publication Type] OR "conference paper"[Title/Abstract] OR "conference review"[Title/Abstract])

The search query for EMBASE was:

('dual energy computed tomography'/exp OR 'dual energy ct' OR 'dual energy computed tomography') AND ('perfusion'/exp OR 'hypoperfusion' OR 'microperfusion' OR 'perfusion' OR 'perfusion theory') AND ('brain'/exp OR 'brain' OR 'cerebra (brain)' OR 'cerebrum' OR 'encephalon' OR 'cerebrovascular accident'/exp OR 'cva' OR 'accident, cerebrovascular' OR 'acute cerebrovascular lesion' OR 'acute focal cerebral vasculopathy' OR 'acute stroke' OR 'apoplectic stroke' OR 'apoplexia' OR 'apoplexy' OR 'blood flow disturbance, brain' OR 'brain accident' OR 'brain attack' OR 'brain blood flow disturbance' OR 'brain insult' OR 'brain insultus' OR 'brain ischaemic attack' OR 'brain ischemic attack' OR 'brain vascular accident' OR 'cerebral apoplexia' OR 'cerebral insult' OR 'cerebral stroke' OR 'cerebral vascular accident' OR 'cerebral vascular insufficiency' OR 'cerebro vascular accident' OR 'cerebrovascular accident' OR 'cerebrovascular arrest' OR 'cerebrovascular failure' OR 'cerebrovascular injury' OR 'cerebrovascular insufficiency' OR 'cerebrovascular insult' OR 'cerebrum vascular accident' OR 'cryptogenic stroke' OR 'ischaemic cerebral attack' OR 'ischaemic seizure' OR 'ischemic cerebral attack' OR 'ischemic seizure' OR 'stroke') AND ([article]/lim OR [article in press]/lim OR [conference paper]/lim OR [conference review]/lim OR [review]/lim) AND [english]/lim AND [abstracts]/lim.
